# Evidence of off-target probe binding in the 10x Genomics Xenium v1 Human Breast Gene Expression Panel compromises accuracy of spatial transcriptomic profiling

**DOI:** 10.1101/2025.03.31.646342

**Published:** 2025-04-03

**Authors:** Caleb Hallinan, Hyun Joo Ji, Steven L Salzberg, Jean Fan

## Abstract

The accuracy of spatial gene expression profiles generated by probe-based *in situ* spatially-resolved transcriptomic technologies depends on the specificity with which probes bind to their intended target gene. Off-target binding, defined as a probe binding to something other than the target gene, can distort a gene’s true expression profile, making probe specificity essential for reliable transcriptomics. Here, we investigate off-target binding in the 10x Genomics Xenium v1 Human Breast Gene Expression Panel. We developed a software tool, Off-target Probe Tracker (OPT), to identify putative off-target binding via alignment of probe sequences and found at least 21 out of the 280 genes in the panel impacted by off-target binding to protein-coding genes. To substantiate our predictions, we leveraged a previously published Xenium breast cancer dataset generated using this gene panel and compared results to orthogonal spatial and single-cell transcriptomic profiles from Visium CytAssist and 3ʹ single-cell RNA-seq derived from the same tumor block. Our findings indicate that for some genes, the expression patterns detected by Xenium demonstrably reflect the aggregate expression of the target and predicted off-target genes based on Visium and single-cell RNA-seq rather than the target gene alone. Overall, this work enhances the biological interpretability of spatial transcriptomics data and improves reproducibility in spatial transcriptomics research.

## Introduction

Recent advances in high-throughput spatially-resolved transcriptomic profiling technologies have enabled the investigation of gene expression with high spatial resolution within tissues. One such commercially available spatial transcriptomics platform is Xenium from 10x Genomics, a publicly traded company with a market capitalization exceeding $1 billion as of March 2025 (Yahoo Finance 2025). Xenium achieves spatial gene expression profiling at single-cell resolution for targeted genes using a probe-based *in situ* detection approach. 10x Genomics currently offers targeted gene panels with pre-designed probe sets, including their 280-gene Xenium v1 Human Breast Gene Expression Panel. As of December 2024, over 16,000 Xenium consumable reactions have been sold, with each tissue slide profiled costing approximately $5,000, underscoring the platform's widespread use and high commercial value (10x Genomics).

Briefly, Xenium uses padlock probes that include sequences complementary to the RNA of target genes. Once a padlock probe binds to its target, it is ligated and subsequently amplified via rolling circle amplification (RCA). Fluorescently labeled decoder probes then hybridize to the amplified RCA product, enabling the simultaneous detection and decoding of the optical signature, or codeword, specific to each gene in the panel through successive rounds of fluorescence imaging. When combined with cell-segmentation, this approach allows for spatially-resolved single-cell quantification of gene expression.

The accuracy of these gene expression measurements thus relies on the specificity of the probes to bind to their intended target gene. We define off-target binding as when a probe binds to something other than the RNA sequence intended to correspond to the target gene (Figure 1). We note once ligation and RCA occur, the resulting fluorescent signal cannot be distinguished between on-target and off-target binding. As such, off-target binding can distort the quantification of the intended target gene's expression, as the observed expression would represent a combination of the target as well as off-target expression.

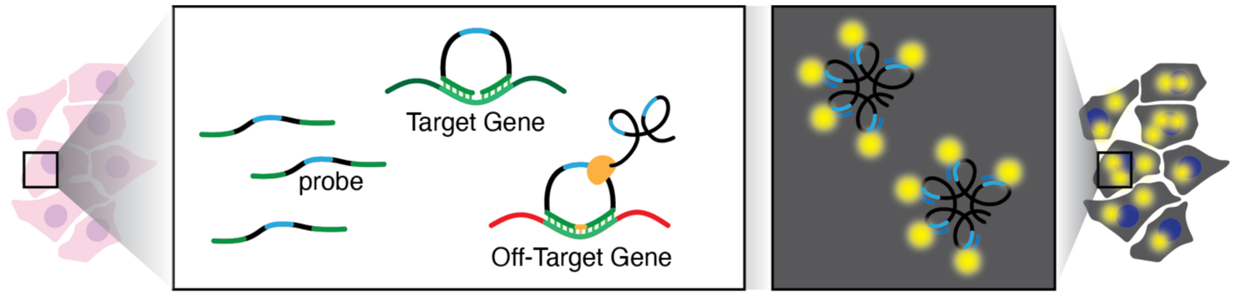

**Figure 1. Schematic of potential off-target binding in 10x Xenium.** In this illustration, the arms of the padlock probes were designed to bind an RNA sequence intended to correspond to a target gene (green). However, these probes exhibit off-target binding and bind to an RNA sequence in a different off-target gene (red). The probe is circularized and subsequently amplified via rolling circle amplification (RCA). Hybridization of fluorescent probes to the RCA product enables the generation a fluorescent signal that is used to quantify RNA expression within cells.

To predict for such potential off-target binding, we developed Off-target Probe Tracker (OPT), a software tool that aligns probe sequences to an annotated transcriptome with the option to allow for mismatches that may still permit probe binding. Using OPT, we identify putative offtarget probe binding to protein-coding genes affecting at least 21 out of 280 genes in the 10x Genomics Xenium v1 Human Breast Gene Expression Panel, compromising the accuracy of their spatial transcriptomic profiles. We substantiate our predictions using data from orthogonal spatial and single-cell gene expression profiling technologies. By facilitating a more rigorous evaluation of probe sequence specificity, tools like OPT can aid in future probe design decisions to help ensure that probes are optimized to minimize off-target binding based on current transcriptome annotations.

## Results

### OPT predicts potential off-target probe binding

To identify potential off-target binding impacting the 10x Genomics Xenium v1 Human Breast Gene Expression Panel, we first downloaded the FASTA file containing the probe sequences from the 10x Genomics Website (Methods, Supplementary Data). This file includes 4,809 probe sequences that are 40bp in length and designed to target 280 genes, averaging 17 probe sequences per gene (ranging from 2 to 50 probe sequences per gene).

We developed a software tool called OPT (Methods) that uses nucmer (Marçais et al. 2018) to predict off-target binding by aligning probe sequences to a reference transcriptome, which comprises a curated collection of transcript isoforms for all genes in a species. OPT features adjustable parameters for binding strictness (e.g., number of mismatches) and generates a summary file that details all targeted genes along with their potential off-targets based on the sequence alignments. 10x Genomics designed its probe sequences using the GENCODE "basic" annotation (Mudge et al. 2025), so initially we also used the latest GENCODE "basic" annotation (v47) to predict off-target binding for these probes.

We first sought to predict if a probe has off-target binding based on perfect sequence homology (i.e., if it aligns with 100% identity) with any annotated transcripts other than those that belong to the intended target gene. Of the 4,809 probe sequences in this gene panel, using GENCODE v47, OPT identified 180 probe sequences across 45 genes as having off-target binding based on perfect sequence homology (Table 1). Among the 45 genes with predicted off-target binding, the number of affected probe sequences per gene ranged from 1 to 14. Overall, these off-target probes matched 90 other genes, including 34 protein-coding genes, 34 pseudogenes, 12 long non-coding RNA, 9 transcripts labeled as nonsense-mediated decay, and one microRNA gene.

| Target gene | Number of probes | Predicted binding genes | Number of probes aligned | Gene types - GENCODE (v47) |
|---|---|---|---|---|
| ADGRE5 | 19 | ADGRE5,ADGRE2 | 19,1 | PC,PC |
| ADH1B | 23 | ADH1B,ADH1A,ADH1C | 22,3,3 | PC,PC,PC |
| AKR1C1 | 21 | AKR1C1,AKR1C5P,AKR1C2,AKR1C3, AKR1C4 | 21,1,1,1,1 | PC,PC,PC,PC,PG |
| AKR1C3 | 17 | AKR1C3,AKR1C1,AKR1C2,AKR1C4 | 17,1,1,2 | PC,PC,PC,PC |
| APOBEC3A | 10 | APOBEC3A,APOBEC3B | 8,4 | PC,PC |
| APOBEC3B | 14 | APOBEC3B,APOBEC3D,ENSG00000284554, APOBEC3F,APOBEC3A | 14,2,2,2,2 | PC,PC,PC,PC,PC |
| AQP1 | 19 | AQP1,ENSG00000250424 | 19,4 | PC,PC |
| C15orf48 | 6 | C15orf48,MIR147B | 6,1 | PC,miRNA |
| C1QA | 4 | C1QA,ENSG00000289692 | 4,2 | PC,PC |
| CD68 | 7 | CD68,ENSG00000264772 | 7,6 | PC,lncRNA |
| CD79B | 5 | CD79B,ENSG00000285947 | 5,3 | PC,PC |
| CEACAM6 | 13 | CEACAM6,ENSG00000267881,CEACAM3 | 13,1,1 | PC,PC,PC |
| CLECL1; CLECL1P | 7 | ENSG00000293488,CLECL1P | 3,3 | lncRNA,PG |
| DPT | 18 | DPT,LINC00970 | 18,13 | PC,lncRNA |
| EPCAM | 15 | EPCAM,ENSG00000225356 | 15,1 | PC,PG |
| FCGR3A | 20 | FCGR3A,FCGR3B,ENSG00000289768 | 20,3,3 | PC,PC,PC |
| HMGA1 | 7 | HMGA1,HMGA1P3,HMGA1P1,HMGA1P2 | 7,1,1,1 | PC,PG,PG,PG |
| IL2RG | 14 | IL2RG,ENSG00000285171 | 14,11 | PC,NMD |
| KLRC1 | 15 | KLRC1,KLRC2,KLRC4-KLRK1,KLRC3, KLRC4,ENSG00000255641 | 15,3,1,1,1, 1 | PC,PC,PC,PC,NMD,PC |
| KRT14 | 6 | KRT14,KRT16P6,ENSG00000290977 | 6,1,1 | PC,lncRNA,PG |
| KRT6B | 15 | KRT6B,KRT6C,KRT75,KRT6A | 15,4,1,1 | PC,PC,PC,PC |
| KRT8 | 18 | KRT8,KRT8P11,KRT8P2,KRT8P3,KRT8P36, KRT8P45,KRT8P33,KRT8P37, ENSG00000304440,CDK5R2-AS1,KRT8P30, KRT8P17, KRT8P22,KRT8P32,KRT8P42 | 18,1,2,3,1, 2, 2,1,1,1,1,1, 1,1,1 | PC,lncRNA,lncRNA,PG,PG,PG, PG,PG,PG,PG,PG,PG,PG,PG,PG |
| LDHB | 18 | LDHB,ENSG00000285854,LDHBP2 | 18,12,1 | PC,NMD,PG |
| LILRA4 | 16 | LILRA4,ENSG00000275210 | 16,3 | PC,lncRNA |
| MYLK | 35 | MYLK,MYLKP1 | 35,1 | PC,PG |
| MYO5B | 20 | MYO5B,MYO5BP1,MYO5BP2, ENSG00000266997 | 20,2,2,5 | PC,PG,PG,NMD |
| PCLAF | 9 | PCLAF,ENSG00000259316 | 9,1 | PC,NMD |
| PDGFRA | 29 | PDGFRA,ENSG00000282278 | 29,9 | PC,PC |
| PTGDS | 5 | PTGDS,ENSG00000284341 | 5,3 | PC,NMD |
| SCD | 20 | SCD,SCDP1 | 20,3 | PC,PG |
| SERHL2 | 8 | SERHL2,SERHL | 8,7 | PC,PG |
| SERPINA3 | 27 | SERPINA3,ENSG00000273259 | 27,14 | PC,NMD |
| SLAMF1 | 24 | SLAMF1,ENSG00000228863 | 23,1 | PC,lncRNA |
| SMS | 17 | SMS,ENSG00000213080,ENSG00000249779, ENSG00000232389,ENSG00-000251342 | 17,7,1,3,1 | PC,PG,PG,PG,PG |
| SVIL | 20 | SVIL,SVIL2P | 20,2 | PC,PG |
| THAP2 | 25 | THAP2,ENSG00000258064 | 25,2 | PC,NMD |
| TPD52 | 21 | TPD52,ENSG00000276418 | 21,5 | PC,NMD |
| TPSAB1 | 2 | TPSAB1,TPSB2,TPSD1 | 2,2,1 | PC,PC,PC |
| TRAF4 | 14 | TRAF4,ENSG00000225869 | 14,1 | PC,PG,PG,NMD |
| TUBA4A | 12 | TUBA4A,TUBA4B | 12,1 | PC,PC |
| TUBB2B | 11 | TUBB2B,TUBB2BP1,TUBB2A,TUBB4A,TUBB3, ENSG00000198211 | 11,2,3,1,1, 1 | PC,PC,PC,PC,PG,PC |
| VOPP1 | 24 | VOPP1,ENSG00000223612 | 24,1 | PC,PG |
| VWF | 20 | VWF,VWFP1,LINC01665 | 20,2,1 | PC,PG,lncRNA |
| ZEB2 | 47 | ZEB2,ZEB2P1 | 47,1 | PC,PG |
| ZNF562 | 22 | ZNF562,ENSG00000291231,ZNF812P | 22,3,3 | PC,lncRNA,PG |

**Table 1. OPT output of genes with predicted off-target binding based on perfect sequence homology in GENCODE v47.** This table shows the 45 genes whose probes in the 10x Genomics Xenium v1 Human Breast Gene Expression Panel exhibit predicted off-target probe binding, where each off-target alignment involves a perfect 40bp match to the probe sequence. Although OPT predicted off-target binding of CCPG1 probe sequences to the DNAAF1-CCPG1 gene, we manually excluded it from our list because DNAAF1-CCPG1 is a read-through gene containing portions of both DNAAF1 and CCPG1. The final column shows the gene types, in order, of each of the off-target genes shown in column 3. Abbreviations: PC = protein-coding; PG = pseudogene; NMD = nonsense-mediated decay; lncRNA = long non-coding RNA.

## Variation in off-target binding predictions across different annotations

OPT relies on alignments to an annotated transcriptome, which ideally reflects all genes and gene variants stably transcribed in a given species. However, genome annotation is still an active area of research (Varabyou et al. 2023), with discrepancies across annotations in gene counts, isoforms, and many other features. We therefore further used OPT to predict for off-target binding and affected genes using two additional human genome annotation sets, RefSeq (v110), (O'Leary et al. 2016) and CHESS (v3.1.3) (Varabyou et al. 2023), and compared to our previous results from GENCODE (v47) (Mudge et al. 2025) (Methods).

When considering only perfect sequence homology, while we previously found 45 affected genes using GENCODE, we found 22 when using RefSeq and 33 when using CHESS (Supplementary Table 1, 2). Given that RefSeq and CHESS have more transcripts than GENCODE, these discrepancies in off-target binding predictions was not simply an artifact of the difference in transcript set sizes.

While the human annotation databases mostly agree on the number of protein-coding genes in the genome, they remain widely divergent on pseudogenes and lncRNA genes. Therefore, we focused on how the results change when we restrict our analysis to only protein-coding genes. By excluding pseudogenes (which are presumably not expressed), lncRNAs, and other non-protein-coding RNAs when using OPT, the number of affected genes fell to 17 for GENCODE and RefSeq and 16 for CHESS (Supplementary Table 3). Again, these discrepancies reflect annotation differences. For example, the probe sequence (ENSG00000196154|S100A4|ab4e3dc), which was designed to target *S100A4* based on GENCODE annotations, also aligns to *S100A5* in RefSeq (Supplementary Figure 1A). We reason that if a probe sequence aligns off-target to a protein-coding gene based on any of these annotations, it could result in off-target binding. We therefore focused further analysis on the union of genes with predicted off-target binding to protein-coding genes across the 3 annotations, resulting in 21 genes: *ADGRE5*, *ADH1B*, *AKR1C1*, *AKR1C3*, *APOBEC3A*, *APOBEC3B*, *AQP1*, *C1QA*, *CD79B*, *CEACAM6*, *FCGR3A*, *FLNB, KLRC1*, *KRT6B*, *PDGFRA*, *S100A4, TOMM7, TPD52, TPSAB1*, *TUBA4A*, *TUBB2B* (Supplementary Table 4).

Comparison with Visium CytAssist reveals spatial gene expression patterns consistent with off-target binding

To investigate the potential effects of our predicted off-target binding for the Xenium v1 Human Breast Gene Expression Panel in experimental settings, we compared spatial gene expression patterns detected in two previously published spatial transcriptomics datasets from serial sections of the same breast cancer tissue: one section assayed with Xenium using the Xenium v1 Human Breast Gene Expression Panel, and another assayed using Visium CytAssist, an orthogonal spatial transcriptomics platform (Janesick et al. 2023). Briefly, Visium is a sequencing-based spatial transcriptomics technology in which RNA is hybridized to spatially barcoded capture spots on a slide, enabling spatial transcriptomic mapping after sequencing. However, while Xenium offers single-cell resolution gene expression quantification, Visium quantifies gene expression within 55μm spots. To enable direct comparison, we first structurally aligned the Xenium and Visium tissue sections using STalign (Clifton et al. 2023), restricting our analysis to overlapping regions since different parts of the tissue were profiled (Supplementary Figure 2A). To enhance visual comparison, we aggregate the Xenium gene expression data at these aligned locations to match the Visium resolution (Supplementary Figure 2B) (Methods).

Among the 21 genes exhibiting off-target binding to protein-coding genes, 12 were present in the Visium dataset that had at least one corresponding off-target gene also detected in the dataset. For genes with no predicted off-target binding based on perfect sequence homology such as *MS4A1*, we observed a visually similar spatial pattern between the two technologies

(Figure 2A), suggesting that spatially aligned groups of cells across the two technologies express this gene at comparable relative magnitudes. However, for genes with predicted off-target binding based on perfect sequence homology such as *TUBB2B*, we observed a visually dissimilar spatial pattern between the two technologies (Figure 2B). Importantly, its predicted off-target gene, *TUBB2A*, in Visium shows a visually more similar spatial pattern to the Xenium *TUBB2B*. To better visualize the effect of off-target binding within the Xenium data, we aggregated the expression of each gene along with its predicted off-targets found in the Visium dataset and visually compared across spatial locations. Notably, the spatial pattern of the aggregated expression of *TUBB2B* and *TUBB2A* in Visium is visually more similar to the spatial pattern of *TUBB2B* in Xenium, consistent with the prediction that Xenium *TUBB2B* probes exhibit off-target binding to *TUBB2A*. We further visually confirmed using the Integrative Genomics Viewer (IGV) that a probe sequence intending to bind to *TUBB2B* also perfectly aligns to a sequence in *TUBB2A*, consistent across all annotations evaluated (Supplementary Figure 1B).

Overall, when comparing the total gene expression between the two technologies, we observed a generally strong positive correlation, consistent with the previously published work (Janesick et al. 2023). We do not observe an obvious trend between gene expression magnitude and the presence of predicted off-target probes (Figure 2C), suggesting that off-target binding prediction alone does not explain the observed higher expression magnitude in Xenium compared to Visium, which may still be attributed to variation in detection efficiency, sequencing depth, and other factors.

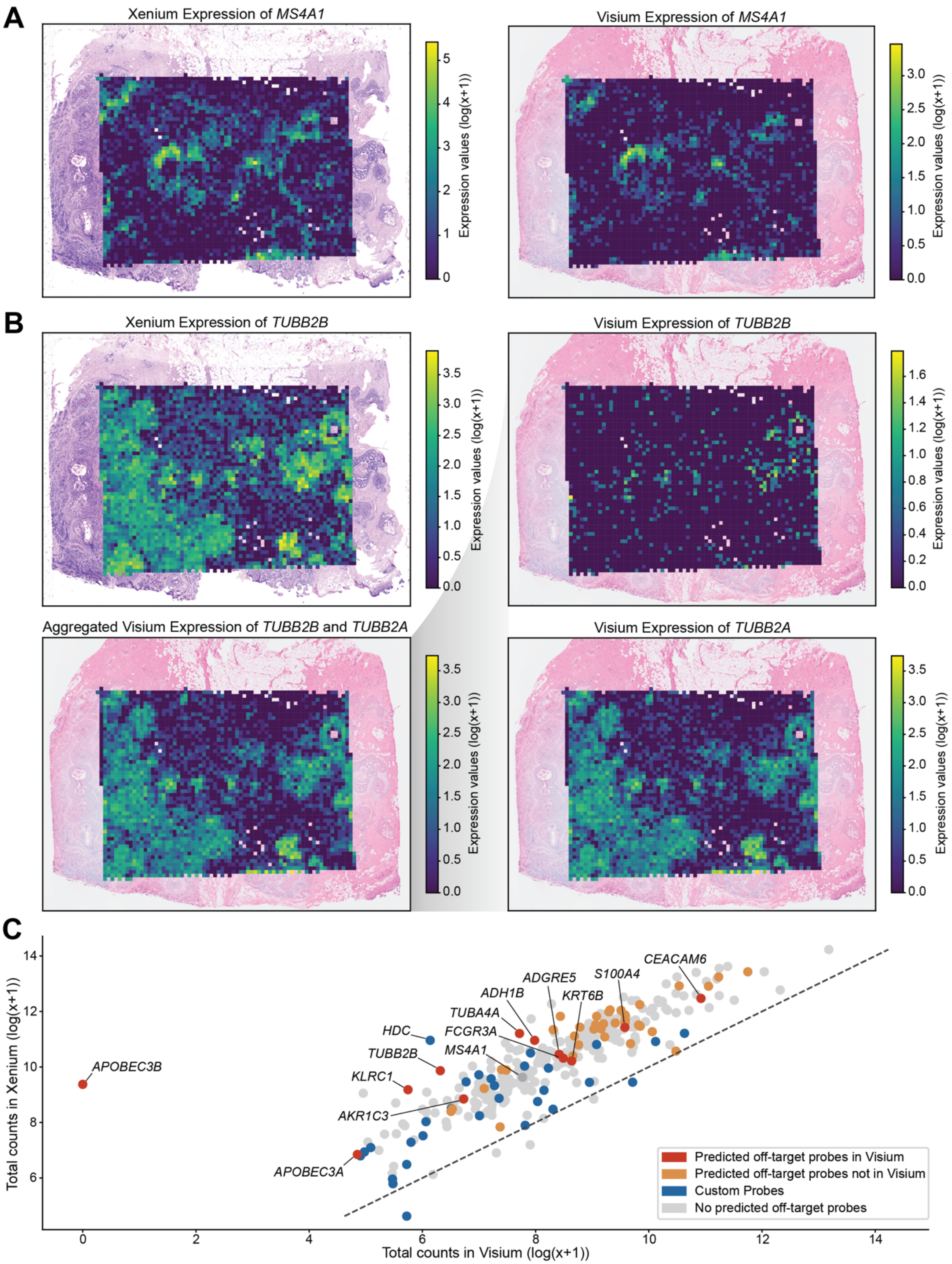

**Figure 2. Comparison of spatial gene expression patterns between Xenium and Visium. (A)** Spatial gene expression of *MS4A1* overlaid on the corresponding histological images for Xenium and Visium. **(B)** Gene expression patterns for *TUBB2B*: Xenium expression, Visium expression, the aggregated Visium expression combining *TUBB2B* and its predicted off-target gene's expression *TUBB2A*, and Visium expression of *TUBB2B*'s predicted off-target *TUBB2A*. (**C)** Scatterplot of log-transformed total expression counts (with a pseudocount) for 307 genes comparing Visium and Xenium data. The dotted line represents X = Y, and points (genes) are colored by probe information.

## Comparison with scRNA-seq reveals single-cell gene expression patterns consistent with off-target binding

To further investigate the potential effects of our predicted off-target binding for Xenium probes, we compared the detected single-cell gene expression patterns in the same previously published work: again, the one assayed with Xenium using the Xenium v1 Human Breast Gene Expression Panel, and another assayed using Chromium Next GEM Single Cell 3' (Janesick et al. 2023). Briefly, single-cell RNA sequencing (scRNA-seq) with 3' end capture is a technique used to profile gene expression at the single-cell level by profiling the 3' ends of mRNA transcripts with sequencing followed by alignment to a genome or transcriptome for quantification. While this approach provides single-cell resolution gene expression quantification, it lacks spatial information. To enable a single-cell comparison with Xenium, we use Harmony (Korsunsky et al. 2019) to remove batch effects and project cells into a shared UMAP embedding (Supplementary Figure 3) (Methods).

Among the 21 genes exhibiting off-target binding to protein-coding genes, 16 were present in the sc-RNAseq dataset that had at least one corresponding off-target gene also detected in the dataset. Again, for genes with no predicted off-target binding based on perfect sequence homology such as *MS4A1*, we observed a visually similar gene expression pattern in the harmonized UMAP across both technologies (Figure 3A), suggesting that transcriptionally similar clusters of cells or cell-types across the two technologies express this gene at comparable relative magnitudes. Likewise, again, for genes with predicted off-target binding based on perfect sequence homology such as *TUBB2B*, we observed a visually dissimilar gene expression pattern on the harmonized UMAP (Figure 3B). Again, its predicted off-target gene, *TUBB2A,* in scRNA-seq showed a visually more similar expression pattern in the harmonized UMAP embedding to the Xenium *TUBB2B*. To better illustrate the impact of the off-target probes, we again aggregated the expression of a gene and its predicted off-target genes present in the scRNA-seq data and visually compared across the harmonized UMAP embedding. The aggregated expression of *TUBB2B* and *TUBB2A* in the scRNA-seq data shows a visually more similar gene expression pattern in the harmonized UMAP embedding to *TUBB2B* in the Xenium, consistent with the prediction that Xenium *TUBB2B* probes exhibit off-target binding with these paralogs (Figure 3B, Supplementary Figure 4).

Overall, when comparing total gene expression between the two technologies, we again observed a generally strong positive correlation (Figure 3C), similar to the Visium comparison results and consistent with the previously published work (Janesick et al. 2023).

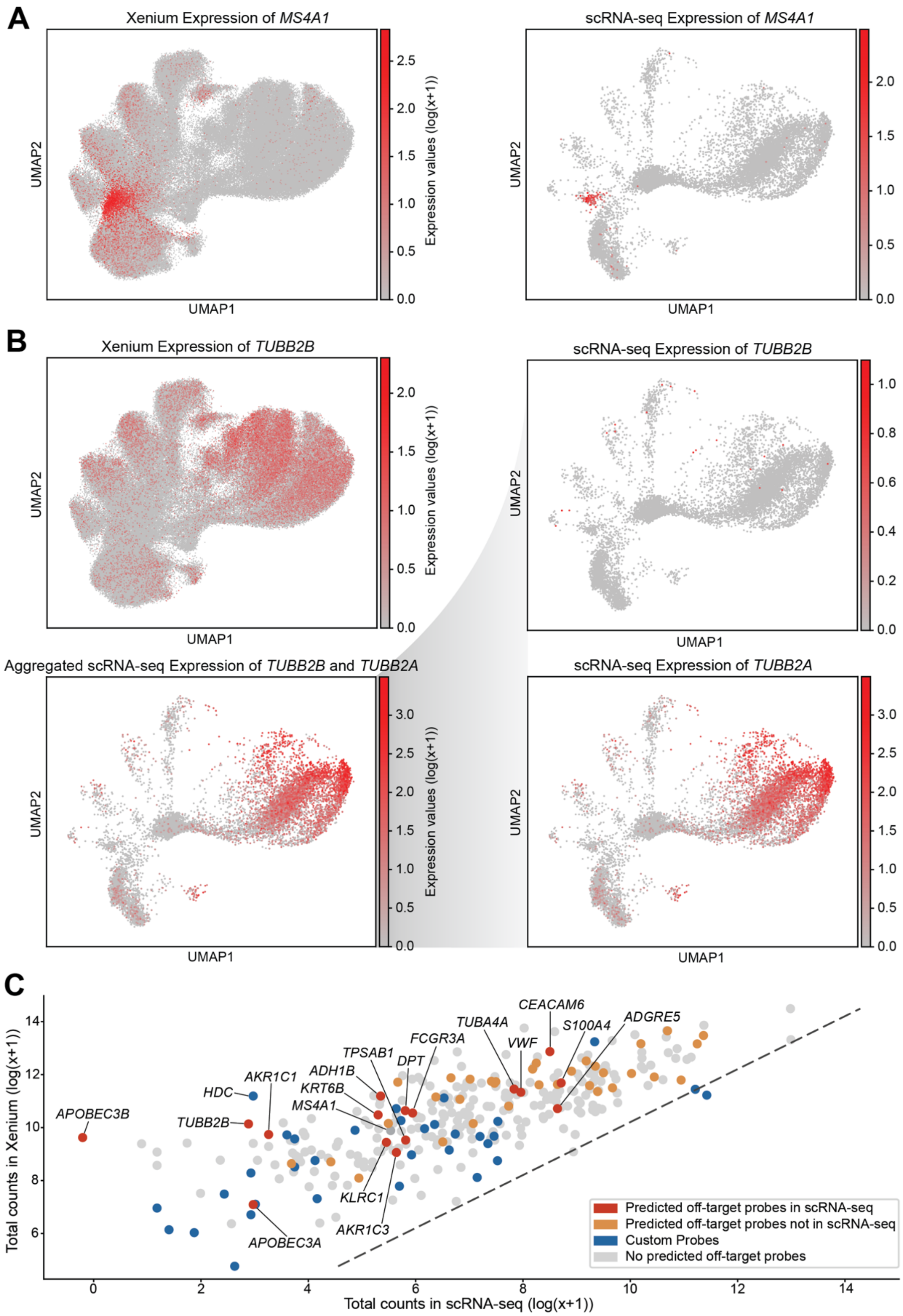

**Figure 3. Comparison of single-cell gene expression patterns between Xenium and scRNA-seq.**

**(A)** Harmonized UMAP visualization of *MS4A1* expression for Xenium and scRNA-seq data. **(B)**

Comparison of *TUBB2B* expression patterns on harmonized UMAP: Xenium expression, scRNA-seq expression, an aggregated scRNA-seq profile combining *TUBB2B* and its predicted off-target gene's expression *TUBB2A*, and scRNA-seq expression of *TUBB2B*'s potential off-target *TUBB2A*. Note that *TUBB2B* also has two additional off-targets present in the scRNA-seq data (Supplementary Figure 4). **(C)** Scatterplot of log-transformed total expression counts (with a pseudocount) for 313 genes between Visium and scRNA-seq data. The dotted line represents X = Y, and points (genes) are colored by probe information.

OPT results when allowing mismatches at the terminal ends of the probe sequences identifies additional off-target candidates

Thus far, we have focused on predicting off-target binding based on perfect sequence homology. However, we reason that imperfect sequence matching could still result in off-target binding. Specifically for the Xenium v1 Human Breast Gene Expression Panel, padlock probes with two 20 base pair (bp) arms bind complementary mRNA regions, forming a 40bp probe sequence. A ligase then circularizes the padlock probe, favoring specific 2-bp junctions. Importantly, if there is a sequence mismatch, particularly outside the ligation site towards the terminal ends of the probe sequence, hybridization may still occur and result in off-target binding, albeit with reduced hybridization efficiency (Supplementary Figure 5A). We therefore added an option in OPT to allow imperfect alignments at the ends of the probe sequences, specifying the sequence length at either ends, where mismatches, insertions, deletions, or clipping can occur (Methods).

Allowing for 6bp mismatches on either end of the 40bp probe sequence_(i.e., requiring a 28bp match covering the middle of the probe sequence including the ligation site) revealed nine additional genes with potential off-target binding when using GENCODE v47 (Supplementary Table 5). Additionally, 18 of the 45 genes previously predicted to be affected by off-target binding based on perfect sequence homology were now predicted to have additional off-target binding, either through the accumulation of new probes with predicted off-target binding or from the identification of additional predicted off-target genes (Supplementary Table 6). Among these additional genes included *CEACAM8*, for which we observed a visually dissimilar spatial pattern between Xenium and Visium (Supplementary Figure 6A). Furthermore, the spatial pattern of the aggregate of *CEACAM8* with its predicted off-target genes (*CEACAM5*, *CEACAM6*, *CEACAM7*, *PSG6*) in Visium more closely resembled the spatial pattern of *CEACAM8* in Xenium. Likewise, a similar trend is observed in the scRNA-seq comparison (Supplementary Figure 6B). Ultimately, these findings suggest that off-target binding, even with imperfect sequence matching, can contribute to the expression patterns observed in Xenium.

## Discussion

Our study presents evidence of off-target probe binding that may distort gene expression profiles affecting at least 21 out of the 280 genes in the Xenium v1 Human Breast Gene Expression Panel. This finding is supported by spatial and single-cell comparative analyses using Xenium with serial section datasets from Visium CytAssist and 3' single-cell RNA-seq respectively. Although we focus here on the Xenium v1 Human Breast Gene Expression Panel from 10x Genomics, we do not exclude the possibility that off-target binding may similarly affect other probe-based gene detection approaches. To assist in the interpretation of existing probe-based gene expression data as well as future probe design, we provide OPT as a software tool for predicting potential off-target probe binding.

Although we have predicted off-target binding based on sequence alignment, its effect on gene expression quantification may still vary. One reason is that the off-target protein-coding or non-protein-coding gene may not be expressed. For example, although *CEACAM6* probes have predicted off-target binding to *CEACAM3* based on perfect sequence homology, the sparse expression of *CEACAM3* in both the Visium and scRNA-seq breast cancer data led to only a minor difference in the aggregated expression (Supplementary Figure 7). As such, the extent of the effect of off-target binding depends on the expression of the off-target gene and will therefore vary across tissues. On the other hand, self-hybridization or interactions between probes themselves may occur to induce non-specific signals (Supplementary Fig 5B). In general, probe binding specificity is influenced by numerous factors, with many methods previously developed to aid in the design of probe sequences while taking these factors into consideration (Wang and Seed 2003; Rouillard, Zuker, and Gulari 2003; Wernersson and Nielsen 2005; Chou 2010; Li et al. 2011; Hu et al. 2020; Hershberg et al. 2021; Fornace et al. 2022).

Notably, our evaluation was limited to the base 280-gene Xenium v1 Human Breast Gene Expression Panel even though the expression of 313 total genes were quantified in these datasets from Janesick et al. This is because 33 of the 313 targeted genes were probed using custom sequences that were not publicly disclosed. Our comparative analysis suggests that *HDC*, a gene with custom probe sequences mentioned in the paper, may also be subject to off-target binding. In Xenium, *HDC* exhibited a distinct spatial pattern and high global expression level, whereas both Visium and scRNA-seq data showed a minimal spatial pattern and sparse expression level respectively (Supplementary Figure 8). We therefore speculate that *HDC* probes may exhibit off-target binding but cannot fully validate without the probe sequences used for *HDC*. As such, we emphasize the importance of publishing full probe sequences to enable reproducible science.

We also observed that 34 / 4,809 breast panel probe sequences did not align to any reference transcripts in the GENCODE v47 annotation. 33 of these unmapped probe sequences matched intronic sequences, which would not normally appear in mature transcripts. We manually aligned the one remaining probe sequence (ENSG00000198851|CD3E|02369ef) to the GRCh38 genome and found that it matched to chr11: 118304577 – 118304616, an intergenic region just upstream of *CD3E*, the target gene for this probe. We suspect that these intron and intergenic panel probe sequences were designed using an earlier version of the GENCODE annotation in which those regions were annotated as exonic. This finding illustrates the importance of disclosing the specific annotation version to promote reproducibility, as well as the ongoing variability of human gene annotation. Likewise, as evidenced by our analysis across GENCODE, RefSeq, and CHESS, we emphasize the variation across these reference annotations and therefore recommend evaluating probe sequences for off-target effects using multiple annotations to be more comprehensive.

Given these challenges, we advise probes with predicted off-target binding to protein-coding genes based on perfect sequence homology be discarded in future experiments. Likewise, we encourage the use of tools like OPT to aid in future probe design decisions and help ensure that probes are optimized to minimize off-target binding based on current transcriptome annotations. For datasets that have already been generated using probes with predicted off-target binding, we generally recommend taking into consideration these predictions to avoid drawing misleading conclusions. For example, we recommend expression measurements for genes with predicted off-target binding be omitted from training foundation models to avoid error propagation. Alternatively, when performing integrative analyses that compare or align gene expression with measurements across technologies, it may be necessary to incorporate off-target binding predictions. For example, integration could be performed between the observed Xenium gene expression and the aggregated expression of the target and predicted off-target genes for the orthogonal technology. Finally, existing literature that base conclusions on genes with predicted off-target binding should be interpreted with caution.

We emphasize that these findings were missed in the previous Janesick et al. publication from 10x Genomics (Janesick et al. 2023). Consistent with previously published observations, we observed a highly correlated total gene expression magnitude between Xenium and Visium as well as scRNA-seq. However, a notable exception is *APOBEC3B*, which is not expressed according to both Visium and scRNA-seq but highly expressed according to Xenium (Supplementary Figure 9) – a discrepancy that Janesick et al. omitted. We emphasize that positive significant average gene expression correlation is a necessary but not sufficient metric for consistency across technologies and that individual data points should be scrutinized. Likewise, validation with orthogonal technologies could have helped identify discrepancies suggestive of off-target effects. We note Janesick et al. only use immunofluorescence to validate two genes, *ERBB2* and *MS4A1*, which by our analysis were predicted to exhibit no off-target binding. We therefore stress the importance of more thorough orthogonal validations. Although Xenium incorporates blank and negative control probes that are intended to help quantify the rate of non-specific and potential off-target binding, our findings suggest that relying solely on such probes for error detection may be insufficient. Implementing probe redundancy, where the same gene is targeted using different codewords, could provide an additional internal control to enable the detection of off-target binding.

This is not the first instance in which a commercially available platform has encountered challenges in probe design (McCartney et al. 2016; Harbig 2005; Mecham et al. 2004; Liu, Bebu, and Li 2014). These findings underscore the critical role of academic researchers towards ensuring the robustness of industry-led product development by providing oversight, free of financial conflicts of interest through independent federal funding. This complementarity between industry and academia fosters a more rigorous, transparent, and reliable scientific process, ultimately to the benefit of consumers and the public. By shedding light on putative off-target probe binding as well as by providing a tool to enable such off-target binding predictions, this work will help enhance the quality of spatial transcriptomics data and improve the overall reproducibility in spatial transcriptomics research.

## Methods

### Off-target Probe Tracker Tool (OPT)

OPT (Off-target Probe Tracker) is a Python program that runs nucmer (Marçais and Kingsford 2011) for alignment and then processes the results to predict probe binding based on sequence homology. OPT is available as an open-source Python toolkit at https://github.com/JEFworks-Lab/off-target-probe-tracker. When a user provides a query probe sequence file, a target transcript sequence file, and the annotation used to extract these transcripts, OPT outputs which gene each probe is likely to bind to. Nucmer is a fast nucleotide sequence aligner that uses maximal exact matches as anchors which it then joins together to find longer alignments. By default, OPT saves nucmer results in SAM format and finds perfect sequence matches between a query probe and a target transcript, requiring that alignments consist of only matches and cover the entirety of the query. OPT consists of three modules: (1) `flip` for reverse complementing probe sequences aligned to the opposite strand of their target genes; (2) `track` for aligning probe sequences and processing alignment results; and (3) `stat` for compiling summary statistics on the number of off-target binding probes and affected genes.

In the case that a probe's target gene has synonyms, we consider alignments to genes annotated with one of its synonym to still be on-target. For example, if a probe that targets *NARS* shows alignments to a gene called *NARS1*, we don't consider it to be off-target binding. We gathered relevant gene synonym relationships using the GeneCards and HGNC online database.

OPT also provides a “pad” mode in which imperfect alignments are allowed at either end of the query (i.e., probe sequence). The -pl parameter sets the pad length at either end of the query and OPT allows for any number of mismatches in these padded regions. For example, if the pad length is 6 and the probe sequence length is 40, then the middle 28bp are the only part of the probe sequence required to match. As long as the critical region is intact, OPT reports an off-target binding site based on this alignment. By default, -pl is set to 0, and the pad mode is activated by providing a non-zero integer to -pl.

Obtaining probe sequences for the Xenium v1 Human Breast Gene Expression Panel

To identify potential off-target binding impacting the 10x Genomics Xenium v1 Human Breast Gene Expression Panel, we obtained the FASTA file of probe sequences from the 10x Genomics Website (https://cdn.10xgenomics.com/raw/upload/v1684948097/software-support/Xenium-panels/hBreast_panel_files/xenium_human_breast_gene_expression_panel_probe_sequences.fasta) and available as Supplementary Data for preservation.

The target gene name and ID was extracted from the probe IDs of the following format:

```
> gene_id|gene_name|accession
```

We expected the provided FASTA file to contain probe sequences to be the reverse complemented sequence of their intended target genes and hence align to the reverse strand of their target isoforms. However, when we aligned the breast panel probe sequences to the GENCODE basic (v47) reference transcripts using nucmer, we found that only 2,508 / 4,809 (52.2%) of probe sequences aligned on the reverse strand of their target transcripts (i.e., isoforms of their target genes). For consistency, we enforced that all probe sequences be oriented in the same direction and align to the forward strand of their target genes and transcripts. As such, we reverse-complemented these 2,508 probe sequences. We also added this functionality as an OPT module called "flip" in which probe sequences aligned to the reverse strand of their targets are reverse complemented. We expect probe sequences to align to the forward strand of transcripts (i.e., both oriented in the same direction) during the downstream probe sequence binding prediction step.

Visium Comparison

The Visium CytAssist dataset, collected from a breast cancer tissue block utilized in Janesick et al., was downloaded from the 10x Genomics website (https://www.10xgenomics.com/products/xenium-in-situ/preview-dataset-human-breast). This dataset originally contained 4,992 spots with x-y coordinates and includes 18,085 genes per spot. Six genes (*AKR1C1*, *ANGPT2*, *BTNL9*, *CD8B*, *POLR2J3*, *TPSAB1*) were excluded from the analysis since they were not present in the Visium dataset.

To compare spatial gene expression patterns from Visium and Xenium technologies, we first mapped all the data to the same coordinate space. We used STalign (v1.0.1), a computational tool that utilizes affine transformations along with diffeomorphic metric mapping to align target and source datasets (Clifton et al. 2023). The initial alignment involved only affine transformations and eight manually determined landmarks to align the Visium histology image (source) to the Xenium histology image (target). This transformation brought the Visium image into the coordinate space of the higher-resolution Xenium image. We then applied this learned transformation to the Visium spots, ensuring that they were correctly positioned relative to both histology images. Next, we used STalign to map the Xenium transcripts (source) onto their corresponding Xenium histology image (target) using both affine and diffeomorphic metric mapping. The transcripts were rasterized at 30μm resolution, with an initial affine transformation guided by four manually defined landmarks. Diffeomorphic metric mapping was then performed with the following parameters: a = 2500, epV = 1, niter = 2000, sigmaA = 0.11, sigmaB = 0.10, sigmaM = 0.15, sigmaP = 50, muA = [1, 1, 1], muB = [0, 0, 0], with all other settings left at their defaults. We extracted the overlapping regions between the two datasets (Supplementary Figure 2A), which reduced the total spots in the Visium dataset to 3,958. Finally, we aggregated the Xenium gene expression data to ~55μm x 55μm patches that correspond to the spatial locations of the Visium spots, resulting in matched-resolution spatial gene expression for both technologies (Supplementary Figure 2B).

Single-cell RNA-seq Comparison

The Chromium Next GEM 3' scRNA-seq dataset, collected from a breast cancer tissue block utilized in Janesick et al., was downloaded from the 10x Genomics website (https://www.10xgenomics.com/products/xenium-in-situ/preview-dataset-human-breast). This dataset contained 12,388 cells with 36,601 genes per cell. All 313 unique genes present in the Xenium dataset are also in the scRNA-seq data, hence both datasets were subsetted to these genes for this analysis.

Both scRNA-seq and Xenium provide single-cell resolution data. To integrate these datasets, we first removed cells lacking detectable gene expression. We the normalized the combined gene expression data using counts per million (CPM) and applied a log transformation with a pseudocount of 1. Principal component analysis (PCA) is then applied to the normalized data, and batch effects are corrected using Harmony (v1.2.3) on the top 30 principal components (PCs) using default parameters except for theta, which was set to 8, to promote further mixing with clusters across technologies. Finally, Uniform Manifold Approximation and Projection (UMAP) is performed on the harmonized PCs, generating a shared 2D embedding across the two technologies, and the data is further facetted by technology for visualization (Supplementary Figure 3).

Cross-annotation analysis

To compare OPT's results with different reference annotations, we used the most recent releases of GENCODE basic (v47), GENCODE comprehensive (v47), RefSeq (v110), and CHESS (v3.1.3) annotation of the GRCh38 genome. Note that GENCODE "basic" is the more reliable version of the annotation and is much closer to RefSeq and CHESS. GENCODE "comprehensive" includes hundreds of thousands of low-quality annotations, which we included in some of our analyses for completeness. Note also that GRCh38 has many non-reference sequences called "alternative scaffolds"; we removed these for our analysis. We then used gffread to extract transcripts as defined in these annotations by running:

```
$ gffread -w transcripts.fa -g grch38.p12/14.fa annotation.gff
```

The GRCh38.p14 assembly was used during transcript sequence extraction for all reference annotations, except for CHESS which specifies that the annotation maps genes and transcripts onto the GRCh38.p12 assembly. For RefSeq, we renamed the VD(J) segment features as transcript features to ensure consistency, and we also removed transcript sequences with the gene_biotype "pseudogene." RefSeq has a separate biotype called "transcribed_pseudogene,"

but doesn't annotate transcripts for these features. We considered transcripts annotated for a small subset of just pseudogenes an error in the annotation.

**Conflicting interests**

The authors declare no conflicting interests.

**Acknowledgements**

We thank Reza Kalhor for his input on the project and feedback on the manuscript. Research reported in this publication was supported by the National Institute of General Medical Sciences of the National Institutes of Health under Awards R35-GM142889 and R35-GM130151, and the HuBMAP Integration, Visualization, and Engagement (HIVE) Initiative under Award Number OT2-OD033760.

## Supplementary Figures

**Supplementary Figure 1. (A)** Screenshot from the Integrated Genome Viewer (IGV) showing the 40bp probe sequence (ID: ENSG00000196154|S100A4|ab4e3dc) that matches both *S100A5* and *S100A4*. Shown are 6 isoforms from the CHESS v3.1 annotation, 4 from GENCODE Basic v47, and 3 from RefSeq v110 for *S100A4*, as well as 2 RefSeq isoforms for the neighboring *S100A5*. The probe sequence aligns to the overlapping region between *S100A5* and *S100A4* gene loci. Matching probe shown in a zoomed-in view below. The forward- and reverse-strand sequences of the probe are shown, and the highlighted area indicates approximately where the probe falls within the gene. **(B)** Screenshot from the Integrated Genome Viewer (IGV) showing the 40bp probe sequence (ENSG00000137285|TUBB2B|1dec8c0) that matches both *TUBB2A* and *TUBB2B*. Shown are the 2 isoforms of *TUBB2A* from the RefSeq v110 annotation, 3 isoforms from the CHESS v3.1 annotation, and 9 from GENCODE Basic v47. Matching probe shown in a zoomed-in view below. The forward- and reverse-strand sequences of the probe are shown, and the highlighted area indicates approximately where the probe falls within the gene. **(A)** and **(B)** share a figure legend.

**Supplementary Figure 2. (A)** Overlap regions between Visium (orange outline) and Xenium (blue outline) data, shown on the Xenium histological image and the Visium histological image, respectively. **(B)** Log transformed aggregated total gene counts for spots (~55$\mu$m x 55$\mu$m) in both Xenium and Visium datasets, overlaid on their corresponding histological image.

**Supplementary Figure 3.** UMAP visualization of integrated scRNA-seq and Xenium datasets: **(A)** before harmony batch correction and **(B)** after harmony batch correction.

**Supplementary Figure 4.** Harmonized UMAP visualization of *TUBB3* and *TUBB4A* expression in the scRNA-seq dataset, as well as the aggregated scRNA-seq expression of *TUBB2B* and all predicted off-targets (*TUBB2A*, *TUBB3*, *TUBB4A*).

**Supplementary Figure 5. (A)** Schematic illustrating that hybridization may still occur even when there is a sequence mismatch at the non-ligated ends of the probe sequence. **(B)** Schematic depicting how probes could bind to each other instead of to their intended target.

**Supplementary Figure 6. (A)** Spatial gene expression patterns for *CEACAM8*: Xenium expression, Visium expression, the aggregated Visium expression combining *CEACAM8* with all predicted off-target binding gene expression, and Visium expression of *CEACAM8*'s potential off-targets (*CEACAM5*, *CEACAM6*, *CEACAM7*, and *PSG6*). **(B)** Comparison of *CEACAM8* expression patterns on harmonized UMAP: Xenium expression, scRNA-seq expression, an aggregated scRNA-seq profile combining *CEACAM8* with all predicted off-target binding gene expression, and scRNA-seq expression of *CEACAM8*'s potential off-targets (*CEACAM5*, *CEACAM6*, *CEACAM7*, and *PSG6*).

**Supplementary Figure 7. (A)** Spatial gene expression patterns for *CEACAM6*: Xenium expression, Visium expression, the aggregated Visium expression combining *CEACAM6* with all predicted off-target binding gene expression, and Visium expression of *CEACAM6* potential off-target (*CEACAM3*). **(B)** Comparison of *CEACAM6* expression patterns on harmonized UMAP: Xenium expression, scRNA-seq expression, an aggregated scRNA-seq profile combining *CEACAM6* with all predicted off-target binding gene expression, and scRNA-seq expression of *CEACAM6*'s potential off-target (*CEACAM3*).

**Supplementary Figure 8. (A)** Spatial gene expression of *HDC* overlaid on the corresponding histological images for Xenium and Visium. **(B)** Harmonized UMAP visualization of *HDC* expression for Xenium and scRNA-seq data.

**Supplementary Figure 9. (A)** Spatial gene expression patterns of: *APOBEC3B* Xenium expression, *APOBEC3B* Visium expression, *APOBEC3F* Visium expression, the aggregated Visium expression combining *APOBEC3B* with all predicted off-target binding gene expression (*APOBEC3F, APOBEC3A, APOBEC3D*), *APOBEC3A* Visium expression, and *APOBEC3D* Visium expression. **(B)** Harmonized UMAP visualization of: *APOBEC3B* Xenium expression, *APOBEC3B* scRNA-seq expression, *APOBEC3F* scRNA-seq expression, the aggregated scRNA-seq expression combining *APOBEC3B* with all predicted off-target binding gene expression (*APOBEC3F, APOBEC3A, APOBEC3D*), *APOBEC3A* scRNA-seq expression, and *APOBEC3D* scRNA-seq expression.

## Supplementary Tables

| Target gene | Number of probes | Predicted binding genes | Number of probes aligned | Gene types |
|---|---|---|---|---|
| ADGRE5 | 19 | ADGRE5,ADGRE2 | 18,1 | PC,PC |
| ADH1B | 23 | ADH1B,ADH1A,ADH1C | 22,3,3 | PC,PC,PC |
| AKR1C1 | 21 | AKR1C1,AKR1C2,AKR1C3,AKR1C4 | 20,1,1,1 | PC,PC,PC,PC |
| AKR1C3 | 17 | AKR1C3,AKR1C1,AKR1C2,AKR1C4 | 16,1,1,2 | PC,PC,PC,PC |
| APOBEC3A | 10 | APOBEC3A,APOBEC3B | 7,4 | PC,PC |
| APOBEC3B | 14 | APOBEC3B,APOBEC3A, APOBEC3D,APOBEC3F | 13,2,2,2 | PC,PC,PC,PC |
| C15orf48 | 6 | C15orf48,MIR147B | 6,1 | PC, precursor_RNA |
| CEACAM6 | 13 | CEACAM6,CEACAM3 | 13,1 | PC,PC |
| FCGR3A | 20 | FCGR3A,FCGR3B | 20,3 | PC,PC |
| FLNB | 33 | FLNB,LOC124909485 | 33,1 | PC,PC |
| KLRC1 | 15 | KLRC1,KLRC2,KLRC3, KLRC4-KLRK1,KLRC4 | 15,3,1,1,1 | PC,PC,PC,PC,PC |
| KRT6B | 15 | KRT6B,KRT6A,KRT6C, KRT75 | 15,1,4,1 | PC,PC,PC,PC |
| S100A4 | 5 | S100A4,S100A5 | 3,1 | PC,PC |
| SERHL2 | 8 | SERHL2,SERHL | 8,7 | PC,misc_RNA |
| SLAMF1 | 24 | SLAMF1,LOC107985220 | 23,1 | PC,ncRNA |
| SVIL | 20 | SVIL,SVIL2P | 20,2 | PC,misc_RNA |
| TOMM7 | 10 | TOMM7,LOC107986346 | 3,2 | PC,PC |
| TPD52 | 21 | TPD52,TPD52-MRPS28 | 21,5 | PC,PC |
| TPSAB1 | 2 | TPSAB1,TPSB2,TPSD1 | 2,2,1 | PC,PC,PC |
| TUBA4A | 12 | TUBA4A,TUBA4B | 12,1 | PC,PC |
| TUBB2B | 11 | TUBB2B,TUBB2A,TUBB3, TUBB4A | 11,3,1,1 | PC,PC,PC,PC |
| ZNF562 | 22 | ZNF562,ZNF812P | 22,3 | PC,misc_RNA |

**Supplementary Table 1.** OPT output of genes with predicted off-target binding based on perfect sequence homology in RefSeq. This table shows the 22 genes whose probes in the Xenium breast cancer panel exhibit predicted off-target probe binding, where each off-target alignment involves a perfect 40bp match to the probe sequence. The final column shows the gene types, in order, of each of the off-target genes shown in column 3. Abbreviations: PC = protein-coding; PG = pseudogene; precursor_RNA = precursor RNA; misc_RNA = miscellaneous RNA; ncRNA = non-coding RNA.

| **Target gene** | **Number of probes** | **Predicted binding genes** | **Number of probes aligned** | **Gene types** |
|---|---|---|---|---|
| ADGRE5 | 19 | ADGRE5, ADGRE2 | 19,1 | PC,PC |
| ADH1B | 23 | ADH1B,ADH1A,ADH1C | 22,3,3 | PC,PC,PC |
| AKR1C1 | 21 | AKR1C1,AKR1C2,AKR1C3, AKR1C4 | 20,1,1,1 | PC,PC,PC,PC |
| AKR1C3 | 17 | AKR1C3,AKR1C1,AKR1C2, AKR1C4 | 17,1,1,2 | PC,PC,PC,PC |
| APOBEC3A | 14 | APOBEC3B,APOBEC3A,APOBEC3D, APOBEC3F | 14,2,2,2 | PC,PC,PC,PC |
| APOBEC3B | 10 | APOBEC3A,APOBEC3B | 8,4 | PC,PC |
| C15orf48 | 6 | C15orf48,MIR147B | 6,1 | PC,miRNA |
| CEACAM6 | 13 | CEACAM6,CEACAM3 | 13,1 | PC,PC |
| EPCAM | 15 | EPCAM,LOC132386 | 14,1 | PC,PG |
| FCGR3A | 20 | FCGR3A,FCGR3B | 20,3 | PC,PC |
| FLNB | 33 | FLNB,LOC124909485 | 33,1 | PC,PC |
| HMGA1 | 7 | HMGA1,HMGA1P1,HMGA1P2, HMGA1P3,HMGA1P6 | 7,1,3,2,2 | PC,PG,PG,PG, PG |
| KLRC1 | 15 | KLRC1,KLRC2,KLRC3,KLRC4-KLRK1,KLRC4 | 15,3,1,1,1 | PC,PC,PC,PC, PC |
| KRT14 | 6 | KRT14,KRT16P6 | 6,1 | PC,PG |
| KRT6B | 15 | KRT6B,KRT6A,KRT6C,KRT75 | 15,1,4,1 | PC,PC,PC,PC |
| KRT8 | 18 | KRT8,KRT8P36,KRT8P11,KRT8P, KRT8P2,KRT8P33,KRT8P45, KRT8P37,KRT8P30,KRT8P17, KRT8P22,KRT8P32,KRT8P42 | 9,1,1,3,2,2, 2,1,1,1,1,1,1 | PC,PG,PG,PG, PG,PG,PG,PG, PG,PG,PG,PG, PG |
| LDHB | 18 | LDHB,LDHBP2 | 18,1 | PC,PG |
| MYLK | 35 | MYLK,MYLKP1 | 29,1 | PC,PG |
| MYO5B | 20 | MYO5B,MYO5BP1,MYO5BP2, LOC102724787 | 20,2,3,2 | PC,PG,PG,PG |
| SCD | 20 | SCD,SCDP1 | 20,6 | PC,PG |
| SERHL2 | 8 | SERHL2,SERHL | 8,7 | PC,PG |
| SMS | 17 | SMS,LOC646347,LOC100419037, LOC642590 | 17,8,1,3 | PC,PG,PG,PG |
| SVIL | 20 | SVIL,SVIL2P | 20,2 | PC,PG |
| TOMM7 | 10 | TOMM7,LOC107986346 | 3,2 | PC,PC |
| TPD52 | 21 | TPD52,TPD52-MRPS28 | 21,5 | PC,PC |
| TPSAB1 | 2 | TPSAB1,TPSB2,TPSD1 | 2,2,1 | PC,PC,PC |
| TRAF4 | 14 | TRAF4,LOC100420513 | 14,1 | PC,PG |
| TUBA4A | 12 | TUBA4A,TUBA4B | 12,1 | PC,PC |
| TUBB2B | 11 | TUBB2B,TUBB2A,TUBB3, TUBB4A | 11,3,1,1 | PC,PC,PC,PC |
| VOPP1 | 24 | VOPP1,LOC729086 | 22,16 | PC,PG |

| | | | | |
|---|---|---|---|---|
| VWF | 20 | VWF,VWFP1 | 20,2 | PC,PG |
| ZEB2 | 47 | ZEB2,ZEB2P1 | 28,1 | PC,PG |
| ZNF562 | 22 | ZNF562,ZNF812P | 22,3 | PC,PG |

**Supplementary Table 2.** OPT output of genes with predicted off-target binding based on perfect sequence homology in CHESS. This table shows the 33 genes whose probes in the Xenium breast cancer panel exhibit predicted off-target probe binding, where each off-target alignment involves a perfect 40bp match to the probe sequence. The final column shows the gene types, in order, of each of the off-target genes shown in column 3. Abbreviations: PC = protein-coding; PG = pseudogene; miRNA = microRNA.

| *Perfect match* | | **GENCODE Basic** | **GENCODE Comprehensive** | **RefSeq** | **CHESS** |
|---|---|---|---|---|---|
| Number of transcripts | | 158,339 | 387,944 | 181,627 | 158,363 |
| All genes | Off-target probes | 180 | 185 | 60 | 112 |
| | Affected genes | 45 | 45 | 22 | 33 |
| Pseudogenes removed | Off-target probes | 151 | 156 | 60 | 46 |
| | Affected genes | 36 | 36 | 22 | 17 |
| Protein coding genes only | Off-target probes | 58 | 58 | 46 | 45 |
| | Affected genes | 17 | 17 | 17 | 16 |

**Supplementary Table 3.** The number of off-target probes and affected genes (from the set of 280 genes in the Xenium panel)) found when looking for perfect matches between probe sequences and transcripts in four different reference annotations: GENCODE basic, GENCODE comprehensive, RefSeq, and CHESS. Off-target alignments between CCPG1 probes and DNAAF1-CCPG1 were excluded.

| **Gene** | **GENCODE** | **RefSeq** | **CHESS** |
|---|---|---|---|
| ADGRE5 | ✓ | ✓ | ✓ |
| ADH1B | ✓ | ✓ | ✓ |
| AKR1C1 | ✓ | ✓ | ✓ |
| AKR1C3 | ✓ | ✓ | ✓ |
| APOBEC3A | ✓ | ✓ | ✓ |
| APOBEC3B | ✓ | ✓ | ✓ |
| CEACAM6 | ✓ | ✓ | ✓ |
| FCGR3A | ✓ | ✓ | ✓ |
| KLRC1 | ✓ | ✓ | ✓ |
| KRT6B | ✓ | ✓ | ✓ |
| TPSAB1 | ✓ | ✓ | ✓ |
| TUBA4A | ✓ | ✓ | ✓ |
| TUBB2B | ✓ | ✓ | ✓ |
| AQP1 | ✓ | x | x |
| C1QA | ✓ | x | x |
| CD79B | ✓ | x | x |
| FLNB | x | ✓ | ✓ |
| PDGFRA | ✓ | x | x |
| S100A4 | x | ✓ | x |
| TOMM7 | x | ✓ | ✓ |
| TPD52 | x | ✓ | ✓ |

**Supplementary Table 4.** Union set of protein coding genes that OPT predicts to be affected by off-target binding, across three different reference annotations: GENCODE basic and comprehensive, RefSeq, and CHESS. Genes not shared across all four annotations are colored in red.

| Target gene | Number of probes | Binding genes | Number of probes aligned | Gene types – GENCODE (v47) |
|---|---|---|---|---|
| ACTG2 | 18 | ACTBP2,ACTG1P11,ACTG1P2 | 1,2,2 | PG,PG,PG |
| ANKRD30A | 20 | ENSG00000215398, ENSG00000215512, ENSG00000290354 | 1,1,1 | PG,PG,lncRNA |
| APOC1 | 2 | APOC1P1 | 1 | lncRNA |
| CD83 | 20 | CD83P1 | 1 | PG |
| CEACAM8 | 13 | CEACAM5,CEACAM6,CEACAM7, CEACAMP8,PSG6 | 1,1,1,1,1 | PC,PC,PC,PG,PC |
| CXCL5 | 16 | CXCL6 | 1 | PC |
| ENAH | 20 | ENAHP1 | 2 | PG |
| FLNB | 33 | ENSG00000263343 | 1 | PG |
| KRT5 | 13 | KRT6B,KRT75 | 1,1 | PC,PC |

**Supplementary Table 5.** Nine additional genes that were identified to exhibit potential off-target probe binding when allowing for a 6bp error margin on either side of the binding site. Abbreviations: PC = protein-coding; PG = pseudogene; lncRNA = long non-coding RNA.

| Target gene | Number of probes | Additional binding genes | Number of additional probes aligned | Gene types – GENCODE (v47) |
|---|---|---|---|---|
| ADH1B | 22 | ADH1A,ADH1C | 1,2 | PC,PC |
| AKR1C1 | 21 | AKR1C3,AKR1C2 | 1,1 | PC,PC |
| AKR1C3 | 17 | AKR1C2,AKR1C1,AKR1C5P | 1,2,1 | PC,PC,PG |
| APOBEC3A | 8 | APOBEC3D | 1 | PC |
| CEACAM6 | 13 | CEACAM5,CEACAM7,CEACAM8 | 1,1,1 | PC,PC,PC |
| FCGR3A | 20 | FCGR3B,ENSG00000289768 | 8,1 | PC,PC |
| KRT14 | 6 | ENSG00000290977,KRT16P6, ENSG00000290978,KRT16,KRT16P2 | 1,1,1,1,1 | lncRNA,PG, lncRNA,PC,PG |
| KRT6B | 15 | KRT6A,KRT5 | 2,1 | PC,PC |
| KRT8 | 18 | KRT8P32,KRT8P36, ENSG00000235286,GFAP,KRT8P14, KRT8P15,KRT8P31,KRT8P35, KRT8P43,KRT8P46,KRT8P47, KRT8P48,KRT8P49,KRT8P7,KRT8P9 | 1,1,1,1,1, 1,2,1,1,1, 1,1,1,1,1 | PG,PG,PG,PC, PG,PG,PG,PG, PG,PG,PG,PG, PG,PG,PG |
| LDHB | 18 | LDHBP1 | 1 | PG |
| LILRA4 | 16 | ENSG00000299952,LILRA6,LILRB3, LILRP1,LILRP2 | 1,2,2,1,2 | lncRNA,PC,PC, PG,lncRNA |
| MYO5B | 20 | MYO5BP3 | 1 | PG |
| PTGDS | 5 | ENSG00000284341 | 1 | NMD |
| SMS | 17 | ENSG00000213080, ENSG00000232389, ENSG00000251342 | 2,4,1 | PG,PG,PG |
| SVIL | 20 | ENSG00000290952, ENSG00000304099 | 1,1 | lncRNA, lncRNA |
| TUBB2B | 11 | ENSG00000198211,TUBB3,TUBB4A, TUBB4B,TUBB4BP3 | 1,1,1,1,1 | PC,PC,PC,PC,PG |
| VWF | 20 | VWFP1 | 1 | PG |
| ZNF562 | 22 | ENSG00000291231,ZNF812P, ENSG00000226810,ENSG00000267490, ZNF561 | 1,1,1,1,1 | lncRNA,PG, PG,PG,PC |

**Supplementary Table 6**. Eighteen genes that were previously predicted to be affected by off-target binding based on perfect matching now show additional predicted off-target interactions when a 6bp error margin is allowed on either side of the binding site. This effect is observed either through the accumulation of new probes with predicted off-target binding or via the identification of additional predicted off-target genes per probe. Abbreviations: PC = protein-coding; PG = pseudogene; lncRNA = long non-coding RNA.

## Supplementary Data

To access a copy of the Xenium v1 Breast Gene Expression Panel fasta file, open the attached Supplementary Data zip file 'xenium_human_breast_gene_expression_panel_probe_sequences.fasta.zip'
